# Advancing through the blood-brain barrier: mechanisms, challenges and drug delivery strategies

**DOI:** 10.5599/admet.2988

**Published:** 2025-10-19

**Authors:** Ronny Vargas, Noelia Martinez-Martinez, Catalina Lizano-Barrantes, Jorge Andrés Pacheco-Molina, Encarna García-Montoya, Pilar Pérez-Lozano, Josep Mª Suñé-Negre, Carlos Suñé, Marc Suñé-Pou

**Affiliations:** 1 Department of Pharmacy and Pharmaceutical Technology, and Physical Chemistry, Faculty of Pharmacy, University of Barcelona, Barcelona, Spain; 2 Department of Industrial Pharmacy, Faculty of Pharmacy, Universidad de Costa Rica, San José, Costa Rica; 3 Department of Molecular Biology, Institute of Parasitology and Biomedicine “López-Neyra” (IPBLN-CSIC), Granada, Spain; 4 Department of Pharmaceutical Care and Clinical Pharmacy, Faculty of Pharmacy, Universidad de Costa Rica, San José, Costa Rica; 5 Pharmacotherapy, Pharmacogenetics and Pharmaceutical Technology Research Group Bellvitge Biomedical Research Institute (IDIBELL), Barcelona, Spain

**Keywords:** Brain delivery, central nervous system, targeted therapy, lipid nanoparticles

## Abstract

**Background and purpose:**

The delivery of therapeutics to the central nervous system (CNS) remains a major challenge due to the restrictive nature of the blood-brain barrier (BBB), a key evolutionary feature that preserves brain homeostasis. This review seeks to synthesize current knowledge on BBB composition, physiology, and transport mechanisms, and critically analyses drug delivery strategies aimed at overcoming this barrier and enabling effective CNS therapies.

**Approach:**

We conducted a comprehensive narrative review integrating evidence on BBB anatomy, transport and permeability mechanisms, drug delivery optimization strategies, with a particular focus on nanotechnology-based systems, and preclinical evaluation models.

**Key results:**

We highlight how a deeper understanding of BBB architecture and dynamic regulation can inform rational design of targeted strategies. Drug delivery approaches are summarized and compared, with emphasis on the potential of nanotechnology-based platforms to enhance CNS drug delivery. Translational considerations, including scalability, reproducibility, and regulatory requirements, are critically addressed. Major challenges identified include receptor saturation, competition with endogenous ligands, disease-specific variability in BBB permeability, and the limited predictive value of current preclinical models. Emerging tools, such as organ-on-chip (for evaluation) and microfluidic mixing (for manufacturing nanomaterials), offer promising means to improve physiological relevance and accelerate translation.

**Conclusion:**

Progress in BBB research has laid the groundwork for innovative therapies, but significant hurdles remain. Advancing CNS drug delivery will require collaborative work refining transport-targeting mechanisms, developing standardized preclinical models, and integrating fundamental research, applied nanomedicine, and regulatory science to open new opportunities for treating neurological and psychiatric disorders and brain tumours.

## Introduction

Over a period of more than 580 million years, the central nervous system (CNS) has evolved through cellular diversification, specialization, and functional compartmentalization [1-3]. It has been theorized that the emergence of a more complex CNS led to the formation of tissue bundles that regulate the exchange of substances between the brain and the bloodstream, thereby protecting brain tissue from harmful agents [4]. This critical tissue for maintaining brain homeostasis is known as the blood-brain barrier (BBB) [5,6].

The BBB is present in all vertebrates and, in more primitive forms, in some invertebrates [7,8]. While less complex organisms have simple barrier structures, higher primates, especially humans, have developed a highly sophisticated BBB composed of various specialised cell types and extracellular matrix components [9,10]. Its low permeability to foreign substances, conserved throughout evolution, is an evolutionary advantage and a key feature that is established during the early stages of embryonic development [7,11,12].

The concept of the BBB originated in the late 19th century, when Paul Ehrlich observed that systemically administered dyes failed to stain the brain, unlike other organs [13,14]. However, he explained this finding in terms of differences in tissue affinity rather than the presence of a physical barrier [12]. This interpretation was later challenged by Edwin Goldman, who demonstrated that dyes which did not stain the brain via systemic administration did so when injected directly into brain tissue, providing clear evidence for a selective physical barrier [14]. Max Lewandowsky further supported the existence of a barrier by showing that neurotoxins had stronger effects when administered intracerebrally compared to systemic routes, thus reinforcing the concept of a functional barrier [14]. The term BBB was formalized through the work of Lisa Stern and colleagues, who identified the dual role of brain endothelial cells (ECs) in both protecting and metabolically supporting the CNS [15].

The development of electron microscopy in the 20th century enabled the precise localization of the BBB to the ECs of brain capillaries and allowed for detailed characterization of their structure [15,16]. This technological breakthrough significantly advanced the understanding of the BBB’s functional anatomy, including intercellular junctions, communication pathways, and transport mechanisms that regulate CNS homeostasis [6].

**Figure 1.**
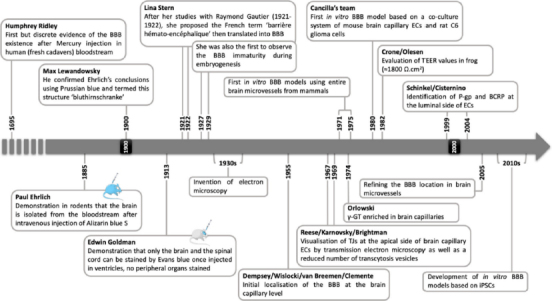
Major research milestones in the development of the BBB concept. Abbreviations: BCRP: breast cancer resistance protein; ECs: endothelial cells; iPSCs: induced pluripotent stem cells; P-gp: P-glycoprotein; TEER: transendothelial electric resistance, γ-GT: γ-glutamyltranspeptidase. Image reproduced from [6] ®CC BY 4.0

Progress in this field led to the adoption of the neurovascular unit (NVU) concept in the early 2000s [10,17], providing a broader framework for understanding the interplay among the components involved in BBB regulation [18]. Figure 1 illustrates how the integration of a growing body of knowledge has improved our understanding of BBB physiology.

However, further research and a deeper understanding of the BBB’s composition are needed to develop effective strategies to overcome BBB permeability, which remains a major challenge to optimizing therapies for neurological diseases [19].

## Blood-brain barrier composition

**Figure 2.**
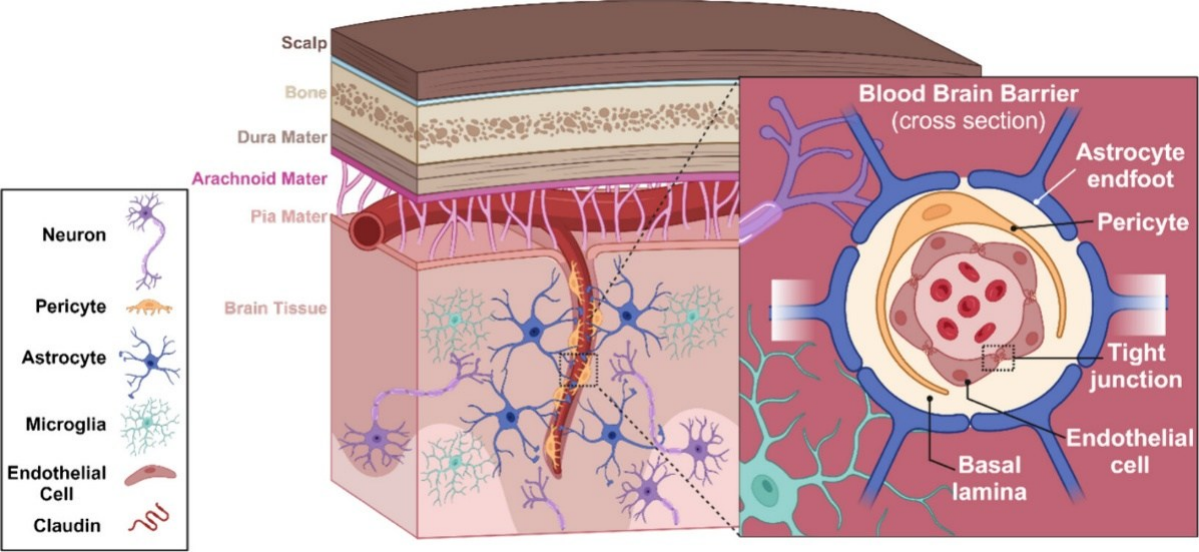
Structure of the neurovascular unit. Tight junctions between endothelial cells restrict paracellular transport, while pericytes partially surround the endothelium. The basal lamina envelops both pericytes and endothelial cells, and is further surrounded by astrocytes, which help maintain barrier properties. Neurons release vasoactive neurotransmitters to regulate blood flow, and microglia contribute to immune function. Reproduced with permission from [25]®

The selective permeability of the BBB is essential for maintaining brain homeostasis, allowing the passage of oxygen and nutrients while restricting the entry of toxins and pathogens [20,21]. This unique property arises from the complex organization of the NVU [10,17,18], which encompasses both the anatomical and functional components of the BBB (Figure 2). The NVU is composed of ECs, pericytes, astrocytes, neurons, myocytes, and the extracellular matrix, all of which dynamically regulate the interactions between the brain and the systemic circulation. Together, these components provide both physical protection and metabolic support to the CNS [10]. Additionally, the NVU acts as a metabolically active interface, exposing penetrant substances to enzymatic degradation or active efflux mechanisms that further enhance its protective role [5,22-24].

### Endothelial cells

Cerebral capillary endothelial cells (ECs) are the principal structural component of the BBB [23,24]. Compared to ECs in other vascular systems, those in the brain exhibit distinct functional and morphological features. First, they have a negatively charged luminal surface that repels polar molecules and exhibit pronounced membrane polarization between the luminal (facing the bloodstream) and the abluminal (facing the CNS) sides, thereby limiting non-specific interactions with circulating substances. Second, cerebral ECs lack fenestrations [26,27], reducing the surface area available for molecular exchange. They are tightly sealed by intercellular junction complexes, comprising tight junctions and adhesion proteins, which restrict the paracellular passage of substances. In addition, these cells display a higher metabolic rate compared to ECs, supporting active, protein-mediated transport systems that regulate selective nutrient uptake and xenobiotic clearance, while exhibiting reduced pinocytic activity [5,26-29].

### Tight junctions

Tight junctions (TJs) between endothelial cells are a key feature of the restrictive permeability of the BBB [24]. These junctions consist of a complex network of proteins that seal the intercellular spaces, including transmembrane proteins (*e.g.* claudins and occludins), cytoplasmic anchoring proteins (such as cingulin and zonula occludens), and cytoskeletal elements [30]. Together, these components form extensive overlapping sealing strands that prevent the paracellular passage of polar molecules and macromolecules into the CNS [31,32].

Beyond acting as physical barriers, TJs also function as lateral diffusion barriers within the endothelial membranes, restricting the redistribution of transporters from the abluminal to the luminal side. This restriction preserves endothelial polarity, thereby maintaining the functional integrity of the BBB [33].

### Pericytes

Pericytes play a fundamental role in the formation of TJs, angiogenesis, and vascular remodelling within the NVU [14,23,32]. They communicate with ECs through cadherin protein-mediated interactions [15,34] and contribute to the expression and polarisation of specific transporters on EC membranes [15]. A reduction in pericyte density has been associated with impaired barrier function [35].

Emerging evidence also implicates pericyte loss in the pathophysiology of various neurodegenerative diseases and neuroinflammatory processes [36]. Their distribution along cerebral capillaries is heterogeneous, varying according to endothelial thickness and capillary diameter [34]. Alongside astrocytes, pericytes regulate vascular tone and function, modulate immune responses, and contribute to metabolic waste clearance [5].

### Astrocytes

Astrocytes are a type of glial cell essential for maintaining the neuronal environment and regulating cerebral signalling dynamics [3,9,37]. They contribute to neurotransmitter homeostasis, waste clearance, cerebral blood flow regulation, ion metabolism, and neuroimmune coordination [5,9].

Strategically located between neuronal synapses and the cerebral vasculature (Figure 2), astrocytes play a central role in the formation and maintenance of the BBB [9,18]. They secrete various factors that modulate the expression of adhesion proteins and endothelial growth factors [38], which are essential for establishing and preserving the selective permeability of the BBB [39,40]. Astrocyte degeneration or dysfunction is recognized as a critical factor in the progression of several neurodegenerative diseases [37].

### Microglia

Microglia, another subtype of glial cells, are central regulators of neuroinflammation and immune surveillance within the CNS. They contribute to brain homeostasis by clearing foreign particles, participating in tissue repair, and mediating extracellular signalling pathways [41]. Their distribution within the brain parenchyma is highly context dependent; for example, microglial density increases in areas adjacent to capillaries in response to inflammation or neuronal damage [42].

Emerging evidence suggests that microglial activity can modulate the expression of TJs proteins, thereby influencing BBB integrity [43]. Given the heterogeneity of microglial subtypes and their multifaceted roles beyond BBB regulation, further investigation is warranted to elucidate their broader impact on CNS physiology and pathology [27].

### Basal lamina

The basal lamina (BL) is the acellular component of the BBB, comprising a specialized network of extracellular matrix proteins that provides structural support and anchorage for the surrounding cellular elements [23,27]. Beyond mechanical stability, the BL is essential for ECs polarization and the regulation of intercellular signalling [43]. This network is mainly composed of collagen, laminin, fibronectin, entactin, and various proteoglycans, which are synthesized and deposited by ECs, pericytes, and astrocytes [27,43]. The composition and structural integrity of the BL are significantly altered in both acute and chronic neuropathological conditions [44,45].

## Endogenous transport mechanisms across the BBB

The brain has a high metabolic demand, requiring various transport pathways to supply essential nutrients and maintain homeostasis [29,46]. Across the BBB, which serves as a dynamic interface that regulates both nutrient influx and metabolic waste removal, essential biomolecules and immune cells are transported to maintain proper brain function and systemic coordination [27,34,47-49].

**Figure 3.**
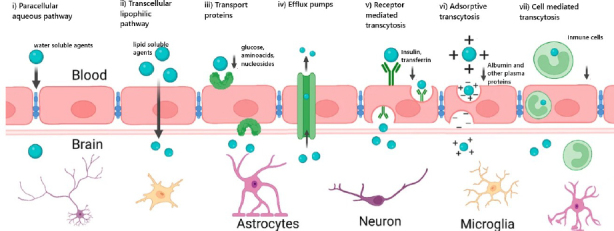
Transport routes across the BBB. Schematic representation of the primary transport mechanisms regulating molecular exchange at the blood-brain barrier. These include (i) paracellular diffusion, (ii) transcellular lipophilic diffusion, (iii) carrier-mediated transport, (iv) efflux pumps, (v) receptor-mediated transcytosis, (vi) adsorptive transcytosis, and (vii) cell-mediated transcytosis. Adapted from [5] CC BY 4.0.

These essential exchanges occur via transport mechanisms broadly classified into two main categories: paracellular diffusion and transcellular transport, which includes passive, carrier-mediated, receptor-mediated, adsorptive-mediated, and cell-mediated transcytosis [29,46]. Figure 3 provides a schematic overview of these transport mechanisms within the NVU. It is important to note that additional endocytic transport systems, which have not yet been fully characterized, are also suggested to contribute to regulating BBB permeability [50].

### Paracellular diffusion

Paracellular diffusion (Figure 3-i) refers to the passive movement of substances through the intercellular space between adjacent ECs [51]. This non-specific transport is driven by concentration gradients from the bloodstream into the brain and is restricted to water-soluble, non-ionised molecules with a molecular weight below 500 Da [29]. TJs play a crucial role in regulating this pathway [27], as structural changes in their composition have been observed to increase the molecular weight threshold for substances crossing the BBB via paracellular diffusion [52].

### Transcellular diffusion

Transcellular diffusion, also known as passive transcytosis (Figure 3-ii), allows the non-specific passage of small, lipophilic, and non-ionised molecules directly across the ECs, driven by concentration gradients [29]. Ethanol and steroid hormones are common examples of molecules utilizing this pathway, which can dissolve into the plasma membrane of ECs [46].

### Carrier-mediated transcytosis

Carrier-mediated transport involves specific transporter proteins, such as GLUT-1 for glucose and LAT transporters for large neutral amino acids [29]. As shown in Figure 3-iii, this mechanism operates when a substrate binds to its transporter on the luminal side of the BBB, inducing conformational changes in the transporter that facilitate its release into the brain parenchyma [46]. This system also mediates the transport of vitamins, organic ions, hormones, nucleosides, and fatty acids [27]. It is an active and selective process that may be ATP-dependent, enabling the movement of substances against concentration gradients [46].

### Receptor-mediated transcytosis

Receptor-mediated transcytosis (Figure 3-v) facilitates the transport of endogenous macromolecules such as insulin, transferrin, leptin, vasopressin and lipoproteins across the BBB [27,51]. Unlike the conformational changes observed in carrier-mediated transport, this mechanism is initiated when a ligand binds to its specific receptor, triggering membrane invagination and the formation of intracellular vesicles known as endosomes. Once internalized, these endosomes undergo acidification, leading to the dissociation of the ligand from its receptor and subsequent exocytosis into the brain [29,46]. This process is mediated by specialized endocytic structures, primarily clathrin- and caveolin-coated vesicles [50,53].

### Adsorptive-mediated transcytosis

Adsorptive-mediated transcytosis (Figure 3-vi), also known as pinocytosis, is a non-specific transport mechanism triggered by electrostatic interactions between positively charged molecules and negatively charged domains on the surface of ECs [50,54]. Although this pathway exhibits lower ligand specificity compared to receptor-mediated transcytosis, it offers a higher transport capacity [46]. It plays a critical role in the transport of certain plasma macromolecules, including albumin and heparin-derived proteoglycans [46,54].

### Cell-mediated transcytosis

Cell-mediated transcytosis (Figure 3-vii) is the most recently described transport mechanism across the BBB [46]. This pathway allows immune cells, including neutrophils, monocytes and macrophages, to cross the BBB under both physiological and pathological conditions [29]. It has been implicated in the infiltration of pathogens, including viruses, fungi and bacteria, into the CNS, earning its designation as the “Trojan horse” mechanism [46,55,56].

### Efflux pumps

Efflux pumps (Figure 3-iv) actively transport molecules out of the brain, preventing their accumulation within the CNS. These ATP-dependent transporters, including P-gp and multidrug resistance proteins [46], play a critical role in maintaining BBB integrity by removing exogenous substances and metabolic products [27]. Efflux can also reduce the effectiveness of certain drugs by limiting the accumulation of compounds that manage to cross the BBB [28].

## Therapeutic limitations imposed by the blood-brain barrier: Implications for central nervous system drug development

The permeability and structural characteristics of the BBB pose significant challenges for drug delivery to the brain. Despite substantial advances in biomedical science, the availability of pharmacological treatments for CNS disorders remains limited due to the inability of many drugs to cross the BBB or to accumulate at therapeutic concentrations within the brain tissue [45,57].

In most cases, the BBB either completely prevents drug accumulation in the brain or requires the administration of excessively high doses to achieve therapeutic concentrations, increasing the risk of systemic toxicity [58,59]. It is estimated that the BBB excludes approximately 98 % of small-molecule drugs and nearly all biomolecules, including proteins, peptides, nucleic acids, antibodies, and viral vectors [27].

CNS disorders place an increasing economic burden on healthcare systems [60,61], currently accounting for approximately 6.3% of the global disease burden [21]. In addition, increasing life expectancy and the resulting ageing of the population suggest that the epidemiological impact of neurodegenerative diseases, which typically manifest between the ages of 50 and 70, will continue to rise. As a result, pressure on healthcare systems is expected to increase, highlighting the urgent need for effective therapeutic strategies to treat CNS disorders [62,63].

Many major pharmaceutical companies allocate limited resources to CNS drug development, primarily due to the high costs, prolonged timelines, and low success rates associated with the clinical translation of these therapies [60,64,65]. Moreover, the development of CNS-targeted therapies is additionally constrained by significant challenges, including an incomplete understanding of the underlying pathophysiology of many neurological disorders, the complexity of defining reliable clinical endpoints, and the formidable barrier posed by the BBB [64].

Developing innovative tools to overcome the challenges of drug delivery to the brain is essential for expanding therapeutic options for CNS disorders [60]. Recognizing the BBB not only as an obstacle but also as a potential path for therapeutic delivery highlights the need for continued research and a deeper understanding of its mechanisms. Harnessing its endogenous transport mechanisms may pave the way for innovative drug delivery systems that improve therapeutic access to the CNS.

## Optimizing therapeutic access to the brain: Strategies to overcome the blood-brain barrier

The development of therapeutic strategies to facilitate drug delivery across the BBB is an area of considerable interest. This research field has greatly benefited from advances in molecular biology and a deeper understanding of both barrier mechanisms and CNS pathophysiology [46].

One approach to CNS drug development involves the synthesis or structural modification of small molecules to enhance their ability to cross the BBB directly. The application of Lipinski's rule of five, or its modifications, has been widely used as a predictive framework for BBB permeability [27,65,66]. According to this rule, a compound is more likely to cross the BBB, mainly by transcellular diffusion, if it meets the following physicochemical criteria: i) molecular weight below 450 Da, ii) partition coefficient (log *P*) between 2 and 4, iii) low hydrogen bond donor capacity (fewer than 10 hydrogen bond donors per molecule), and iv) p*K*_a_ between 6 and 10.5, ensuring minimal ionisation at physiological pH [46,67].

**Figure 4.**
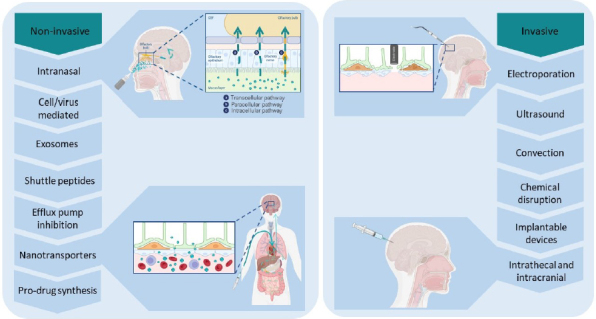
Classification of methods for drug delivery across the BBB. This figure presents some of the most used strategies for delivering therapeutics to the CNS, categorized into non-invasive and invasive approaches. Figure created by the authors with BioRender.com based on information compiled from several references included in this review

However, not all potential therapeutic compounds can be structurally modified to meet these criteria [46,66]. Moreover, studies have shown that over one-third of compounds that successfully cross the BBB do not adhere to Lipinski's rule, and that log *P* values of permeable and non-permeable compounds do not differ significantly [68]. Therefore, relying solely on this approach is insufficient for effective CNS targeting. As a result, alternative strategies have been developed that leverage physical, chemical, and biological mechanisms to enhance drug transport across the BBB [57,61,65,67,69-71]. These strategies can be broadly classified into invasive and non-invasive methods, as illustrated in Figure 4.

### Invasive methods

To overcome the limited accumulation of systemically administered drugs in the CNS, some treatments are delivered directly to brain tissue through either physical or biochemical disruption of the BBB, or by implantation of biodegradable, sustained-release systems [69]. These approaches are particularly viable in the context of neurosurgical interventions following malignant tumour resection, or in the treatment of subarachnoid hemorrhage and traumatic brain injury [60].

### Blood-brain barrier disruption

BBB disruption methods temporarily increase barrier permeability by compromising the integrity of ECs or TJs, using either chemical agents or physical stimuli. Chemical disruption involves the administration of compounds such as mannitol, ethanol, hyperosmotic solutions (*e.g.* dextrose), or specific toxins. Physical disruption methods include radiation, microwaves, thermomagnetic stimulation, and ultrasound [46,66,67,69].

Osmotic opening is widely used in clinical practice; however, it is non-selective and often associated with neurotoxicity [27]. While chemical disruption can be effective, it carries significant drawbacks, as it may compromise the integrity and function of the BBB, potentially allowing harmful substances to enter the CNS [66].

Ultrasound offers an alternative and promising approach by momentarily suppressing the expression of tight junction proteins, thereby increasing BBB permeability without causing direct tissue damage. It has been shown to improve drug penetration into brain tumours [66]. This method is localized, transient, and reproducible. However, it requires specialized equipment, and further research is needed to elucidate the precise mechanisms of BBB opening and to establish optimal monitoring conditions [27]. Some authors classify ultrasound-induced BBB opening as a non-invasive technique [72], primarily due to its reversibility, although this classification remains controversial given its impact on barrier integrity.

Electroporation is a less aggressive invasive alternative for BBB disruption. This technique uses pulsed electric fields to momentarily destabilize cell membranes, thereby increasing permeability in a controlled and reversible manner [69,70].

#### Intracranial administration

This delivery approach bypasses the BBB by introducing drugs directly into the CNS via diffusion through the cerebrospinal fluid. Intrathecal administration involves the injection into the spinal canal or subarachnoid space [69], whereas intracranial administration refers to the direct delivery of drugs into the cranial cavity, including intracerebral, intracranial, or intraventricular injections [72,73].

The main advantage of these methods is that the entire administered dose reaches the brain tissue, avoiding high systemic concentrations that could lead to adverse effects [45,57,66], which is particularly relevant for potent drugs with high systemic toxicity [69,72]. However, these techniques are highly invasive and may result in poor patient compliance, especially for treatments requiring repeated dosing [27].

#### Convection-enhanced delivery

When a drug is administered directly into the brain parenchyma, its distribution occurs primarily by passive diffusion along a concentration gradient [72]. However, this process is limited by the relatively low extracellular fluid volume and slow diffusion rates within brain tissue [60]. Convection-enhanced delivery (CED) overcomes these limitations by applying positive pressure during administration to facilitate drug dispersion [73].

In CED, a catheter is inserted into a brain tumour, and a continuous infusion system maintains a positive pressure gradient to enhance drug penetration [65,67]. Despite its potential benefits, CED presents challenges such as unpredictable drug distribution, difficulty in real-time monitoring, and the need for personalized optimization of catheter design and placement [27].

#### Implantable drug delivery devices

Another invasive technique involves the surgical implantation of biodegradable polymer-based drug delivery systems into the CNS [67]. These devices allow sustained drug release [27] and show promise for the treatment of neurodegenerative diseases and brain injury [21]. Implant types include gels, microspheres, nanospheres, and wafers [65]. Nonetheless, these systems have notable limitations, including restricted drug distribution, dose constraints due to implant size, and unresolved concerns regarding long-term safety [27].

### Non-Invasive methods

Non-invasive approaches primarily rely on pharmaceutical strategies that enable drug delivery to the CNS while avoiding the risks and invasiveness of surgical interventions [66]. Traditional non-invasive methods include intranasal administration, the exploitation of endogenous transport mechanisms, such as viral vectors, extracellular vesicles, and immune cells, and drug modification using transport vectors and nanotechnology-based delivery systems [69,70].

Additionally, the synthesis or structural modification of drugs, including prodrug strategies outlined at the beginning of Section *Optimizing therapeutic access to the brain: Strategies to overcome the blood-brain barrier*, also falls within this category [27,65].

#### Intranasal administration

The nasal route is a drug delivery pathway that can be used for both systemic administration and direct brain access [69]. Through this route, small lipophilic molecules and biological agents can penetrate the nasal epithelium and reach the CNS. Alternatively, drugs can reach the brain via transneuronal transport along the trigeminal and olfactory nerves [60,66]. In recent years, intranasal delivery has been the subject of extensive research, demonstrating efficacy in preclinical models for various therapeutic applications. Current efforts are focused on achieving clinical translation, positioning this strategy as a promising alternative for the treatment of CNS disorders [74-76].

This method is fast and offers high bioavailability once the drug is absorbed. However, bypassing the nasal epithelium and mucus layer, as well as avoiding enzymatic degradation by the nasal mucosa, remains a challenge, making this route particularly suitable for highly potent drugs [27].

The use of nanoparticles (Section *Efflux pump inhibition* below) in this context enhances drug interaction with the nasal epithelium and mucus and prevents the enzymatic degradation. Some examples of nose-to-brain delivery include the study by Koo *et al.* [77], which used polymeric micelles functionalized with a cell-penetrating peptide to target glioblastoma, demonstrating in vivo efficacy. Sharma *et al.* [78] reported enhanced brain concentrations of paroxetine using chitosan-modified polymeric nanoparticles, while Deshmukh *et al.* [79] achieved successful delivery of paroxetine using ligand-conjugated polymeric nanoparticles.

#### Viral vector-mediated drug delivery

Neurotropic viruses can be used as effective drug delivery vehicles to the brain [65]. This can be achieved either by using viral vectors that inherently cross the BBB or by modifying their surface to incorporate natural ligands that interact with endogenous receptor-mediated transcytosis systems [80,81]. Viral vectors can also be combined with hyperosmotic agents to enhance transcellular transport [66]. Common examples of viral vectors include adenoviruses, herpesviruses, recombinant SV-40 viruses, and lentiviruses, among others [27,66].

These vectors offer high efficiency, specific cellular tropism, and relative scalability. However, they also carry risks, including potential immune responses, oncogenesis, and mortality [27].

#### Cell-mediated drug delivery

Cell-mediated drug delivery, also known as the “Trojan horse” strategy (see Section *Cell-mediated transcytosis*), utilizes immune cells such as macrophages, monocytes, and neutrophils to transport therapeutic agents across the BBB [46,65,82]. The phagocytic properties of these cells facilitate the internalization of therapeutic agents and their subsequent transport into the brain during the migration of immune cells in response to inflammatory processes [69].

A practical advantage of this approach is that it does not require drugs to be nanometric in size, as is often required for other strategies [46]. However, limitations include potential issues with cellular viability due to the transported compound and the risk of immune activation, which may lead to adverse effects such as an increased risk of thrombosis [65].

#### Exosome-based drug delivery

Extracellular vesicles, particularly exosomes, are endogenously derived particles ranging in diameter from 30 to 150 nm, with the ability to cross biological barriers [27,66]. These small membrane-derived vesicles can encapsulate and transport lipids, nucleic acids, and proteins. Theoretically, they are produced by all cell types [83] and can be used for drug delivery in either their natural or modified forms [84].

Exosomes have demonstrated the ability to cross the BBB and play a role in peripheral-to-brain communication, contributing to normal neuronal function [85]. They are highly biocompatible and provide protection and stability for the encapsulated therapeutic agents [27].

Some illustrative applications of exosome-based delivery systems include brain-targeted exosomes that are surface-modified with peptides for targeting. For example, exosomes loaded with miR-133b have demonstrated neuroprotective effects in a Parkinson’s disease model by improving motor function, reducing Tau phosphorylation, and attenuating neuronal damage [86]. In traumatic brain injury models, exosomes encapsulating neuroprotective peptides have demonstrated cytoprotective effects in vitro and improved behavioural and histological outcomes in vivo following intravenous administration [87]. Moreover, exosome-based strategies have been used to enhance the delivery of temozolomide for glioblastoma treatment, resulting in increased apoptosis and improved therapeutic efficacy [88].

However, and despite their promising results, several challenges remain, particularly in the standardization of isolation and purification protocols [27]. In addition, a significant proportion of systemically administered exosomes is sequestered by the liver, lung, spleen, and phagocytic cells of the immune system. This issue can be mitigated by surface modification with peptides or proteins to enhance brain targeting [27,66].

#### Shuttle peptides and protein vectors

Peptides can facilitate CNS drug delivery by exploiting the BBB intrinsic transport mechanisms [89]. This strategy involves modifying drugs with cationic proteins or peptides to facilitate their transport across the BBB via endogenous transport mechanisms [46,89]. These vectors, known as shuttle proteins or shuttle peptides, can be used for small molecule drugs [90], biologics [91,92], or combined with other delivery strategies such as nanoparticles [93-95], as explained in the Section N*anotechnology-based strategies for drug delivery across the BBB.* Shuttle peptides allows both passive and active transport across the BBB, enhancing versatility and efficiency in drug delivery [89,90].

#### Efflux pump inhibition

Given their role in limiting drug accumulation within the CNS, efflux pumps have become a target for strategies aimed at improving therapeutic retention in the brain. Selective inhibition of these transporters has been explored to improve drug efficacy, particularly for lipophilic compounds that are otherwise rapidly cleared before reaching therapeutic concentrations [65,67,69].

#### Nanotechnology-based strategies for drug delivery across the BBB

Nanostructures have unique properties that enhance their potential for crossing physiological barriers and support applications in drug delivery, brain imaging and diagnostics. These properties include their small size, surface modification capabilities, material versatility for fabrication, tunable surface characteristics, high drug loading capacity, and controlled drug release [20,46,66,67]. In addition, nanotechnology-based carriers can minimize adverse effects [21] and improve biocompatibility, particularly in the case of lipid nanoparticles (LNPs) [96,97]. Many of these features are also advantageous for industrial-scale manufacturability [98,99].

A wide range of nanocarriers have been investigated for CNS drug delivery, including nanoemulsions, micelles, liposomes, nanofibers, nanorobots, polymeric nanoparticles, solid lipid nanoparticles, and nanostructured lipid carriers [21]. Among these, polymeric and lipid-based nanoparticles (including liposomes) have been the most extensively studied for BBB transport [46].

The use of nanoparticles, particularly LNPs, has increased significantly over the past decade as a recognized non-invasive strategy for enhanced drug delivery across the BBB [19,70,100-103]. Due to their lipid composition, these nanoparticles exhibit a natural propensity for brain uptake, even in the absence of surface modifications for active targeting. However, some authors argue that this passive approach lacks precision and control. As a result, active targeting strategies have gained attention as more promising and reliable methods for BBB penetration [19,93,104,105].

**Figure 5.**
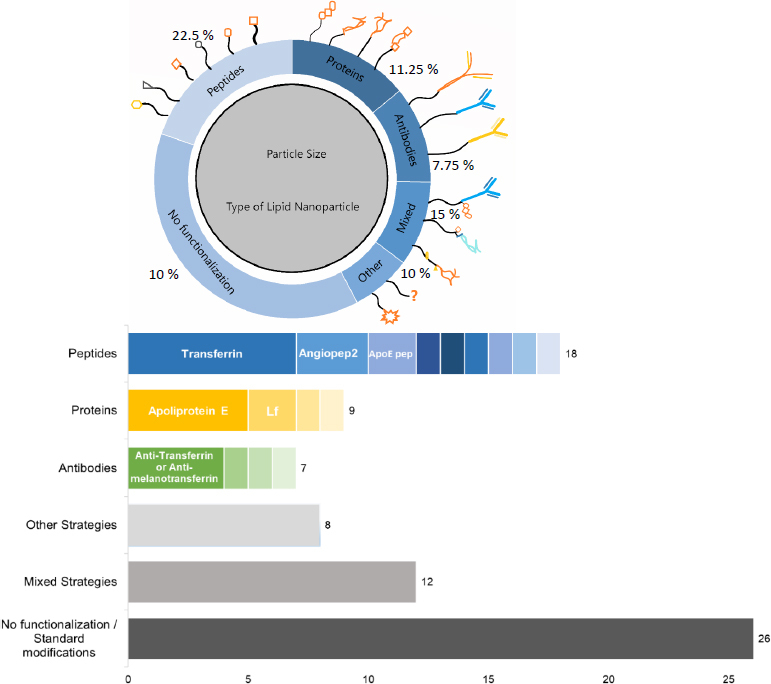
Functionalization strategies for LNPs to cross the BBB. This figure illustrates the main functionalization strategies employed to enhance the transport of LNPs across the BBB. Notable peptides include transferrin, Angiopep-2, and an apolipoprotein E-derived peptide. Among proteins, apolipoprotein E and lactoferrin are the most frequently used, while transferrin and melanotransferrin-targeting antibodies are predominant antibody-based strategies. Adapted from [19] CC-BY-NC-ND

Advances in this area have highlighted peptides, proteins, and antibodies as key strategies for nanoparticle functionalization to enhance BBB penetration [19] via receptor-mediated transcytosis [29]. Other functionalization approaches have been explored, including the use of aptamers [106], neurotransmitter-derived lipids [107], and materials such as menthol [108] or tocopherol derivatives [109]. Combined strategies have also been reported, such as conjugation of peptides with folic acid [110], antibodies with proteins [111], aptamers with peptides [112], or peptides with biomimetic systems. These biomimetic systems provide an additional approach by using cell-mediated transport to improve BBB permeability [113]. Figure 5 illustrates the distribution of functionalization strategies reported between 2016 and July 2024 in studies evaluating the efficacy of functionalized nanoparticles as drug carriers across the BBB, comparing their results with their non-modified counterparts [19].

Current functionalization approaches show a strong preference for peptides as the primary strategy to facilitate nanoparticle permeability across the BBB. These short amino acid sequences provide receptor specificity without the immunogenicity or production complexity of full proteins [104,114,115].

Several peptides have been extensively studied for nanoparticle functionalization, including Angiopep-2, the rabies virus glycoprotein (RVG) peptide, transferrin receptor-targeting peptides, and cell-penetrating peptides [116-120]. Among these, Angiopep-2 was one of the first to be investigated due to its affinity for low-density lipoprotein receptor-related protein 1 (LRP1), a key mediator in lipid transport and brain homeostasis [116,121,122]. The RVG peptide exhibits potent neurotropism through high-affinity binding to the acetylcholine receptor, enabling the first demonstration of peptide-mediated nucleic acid delivery to the CNS in 2007 [118,123].

The transferrin receptor is widely expressed throughout the body, but one of its most notable applications is as a gateway to the CNS [119]. The THR peptide binds specifically to the human transferrin receptor and enhances nanoparticle transport across the BBB, although its stability is limited by its susceptibility to blood proteases. The retro-enantiomeric version (THRre) has been developed to improve serum stability and transport efficiency, representing a third-generation peptide with enhanced BBB penetration [117,118].

Beyond these, other peptides originally studied as transport vectors are now being explored for nanoparticle functionalization. One such example is MiniAp-4, a cyclic peptide derived from apamin, a neurotoxin found in bee venom. MiniAp-4 has been optimized to reduce complexity, toxicity, and immunogenicity while preserving BBB targeting, active transport, and protease resistance. MiniAp-4 represents a promising candidate for safe and efficient nanoparticle-based brain delivery [124].

## Methods for evaluating blood-brain barrier permeability

Successfully translating CNS-targeted therapies into clinical applications requires thorough evaluation. The development and optimization of novel transportation strategies must be accompanied by rigorous permeability assessments to ensure efficient drug delivery to the brain [125,126]. Various models are available, each with different levels of complexity, representativeness, and applicability. The selection of the model depends on available resources and the specific objectives of each study [127,128].

In silico models use computational tools to generate rapid and cost-effective predictions of passive drug transport across the BBB [127,129]. While valuable in early-stage development, they cannot fully replicate the biological, molecular, and mechanical complexity of the BBB [126]. In vitro models, based on primary or immortalized cell cultures, can be performed as monocultures or co-cultures with other BBB components [126,127]. These models are useful for evaluating compound permeability; however, their correlation with in vivo models may be limited due to the simplification of barrier physiology [126,130] In vivo models involve animal studies using primary rodents, zebrafish, and non-human primates [131-133]. These models provide a complete biological system for BBB permeability assessment but raise ethical concerns, involve high costs, and present interspecies differences that complicate translation to human physiology [127].

An emerging alternative is organ-on-a-chip (OoC) technology, which integrates microfluidics and 3D cell cultures to more accurately replicate the architecture and physiological conditions of the in vivo environment [134-136]. These systems offer enhanced cellular differentiation and more physiologically relevant responses, making them highly representative research tools. By recapitulating key barrier functions, OoC models provide a promising bridge between in vitro and in vivo systems for BBB evaluation [136,137]. However, further validation and optimization are still needed to ensure robustness and reproducibility [126].

Regardless of the model used, proper validation is essential to ensure clinical relevance. While a detailed discussion of each method is beyond the scope of this article, readers are encouraged to consult existing reviews for a more comprehensive analysis of their principles, advantages, and limitations. As research on the BBB continues to advance, it lays the groundwork for the development of novel therapeutic strategies.

## Discussion

Drug delivery to the CNS is an interdisciplinary field that requires both incremental and synergistic advances. The complexity of the BBB implies that optimizing brain-targeted therapies demands progress across multiple disciplines, including physiology, materials science, bioengineering, and pharmacology. Successfully bypassing this barrier requires a profound understanding of the BBB, not only its anatomical structure, but also its dynamic modulation, intercellular communication, and transport mechanisms. These processes define the BBB's selective permeability and determine how pharmacological agents interact with and cross the barrier to achieve effective delivery while preserving CNS homeostasis.

The challenges of delivering therapeutics across the BBB are becoming increasingly critical as recent therapeutic strategies shift from small molecules to biological macromolecules and nucleic acid-based approaches, which face even greater obstacles for entry into the CNS. The growing interest in RNA-based therapeutics and gene modulation strategies for neurological disorders exemplifies this paradigm shift and underscores the urgent need for innovative delivery approaches to ensure effective brain targeting.

This review has compiled and analysed a wide range of strategies to overcome the BBB, from passive diffusion enhancers to active targeting mechanisms involving ligand functionalization. A comparative analysis of these approaches reveals key patterns in their effectiveness and limitations, providing valuable insights into their translational potential. Particular attention has been given to nanotechnology-based systems, especially LNPs, due to their versatility and promise in CNS drug delivery.

LNPs have emerged as a relatively safe and versatile platform, owing to their structural resemblance to biological membranes. They provide protection and controlled release of therapeutic agents and can be surface-functionalized to enhance interactions with biological barriers. Functionalization strategies—such as those involving Angiopep-2 and transferring receptor-targeting peptides— have demonstrated potential to improve BBB permeability. However, these approaches also pose challenges, including receptor saturation, competition with endogenous ligands, and variability in receptor expression across disease states. Achieving an optimal balance between targeting specificity and clinical applicability remains a critical challenge.

Despite the promise of these strategies, most remain at the preclinical or laboratory scale, highlighting the need for further research to bridge the existing gap and facilitate their translation into clinical practice.

Observations on BBB permeability models also highlight the need for a paradigm shift in preclinical testing. Traditional in vitro models provide valuable insights, but often lack the complexity of the in vivo environment, resulting in limited correlation with in vivo results. Conversely, in vivo models raise ethical concerns and may fail to reflect human physiology accurately. The emergence of OoC platforms offers a promising middle ground, enabling human-relevant, high-throughput screening of nanoparticle behaviour under physiologically relevant conditions. However, most OoC systems remain at the proof-of-concept stage, requiring further validation and a robust regulatory framework before they can be fully integrated into preclinical pipelines.

In light of these advancements and ongoing challenges, the field of BBB drug delivery remains a critical frontier in biomedical research. The convergence of nanotechnology, biomolecular engineering, and emerging preclinical models offers unprecedented opportunities to enhance CNS-targeted therapies. However, significant hurdles still need to be overcome to enable the clinical translation of these strategies. Addressing these challenges will be essential for advancing the treatment of neurological and psychiatric disorders, as well as brain tumors, thus paving the way for more effective and targeted therapeutic interventions.

### Challenges and future directions

Despite decades of research, significant uncertainties remain regarding the roles of specific components of the NVU. A more comprehensive understanding of their function could unveil previously unrecognized pathways for drug transport, thereby expanding the landscape of BBB-targeting strategies. Key areas for future investigation include glial cell functions, the molecular mechanisms governing BBB integrity, and the dynamic, context-dependent modulation of its transport systems, particularly endocytic pathways. Additionally, advancing pathological insights into neurological diseases could yield valuable clues for optimizing therapeutic interventions. In this context, personalized nanomedicine represents a promising avenue, with patient-specific factors offering the development of precision therapeutics tailored to individual needs.

The diversity of potential approaches for CNS drug delivery underscores the growing need for research to ensure both safety and efficacy. Each delivery strategy presents distinct advantages and limitations, just as each neurological disorder is characterized by a unique pathophysiology. Although many of these innovative strategies demonstrate considerable promise, most remain at the preclinical or early investigational stages and have yet to transition into clinical application. Advancing in this field requires an integrated, multidisciplinary approach—leveraging emerging technologies, combining complementary strategies, refining delivery systems, and aligning interventions with the molecular characteristics of specific diseases—to develop effective, scalable, and patient-centered therapeutics for CNS disorders.

Furthermore, the development of robust evaluation methods to assess the effectiveness of these systems in crossing the BBB is crucial. Ethically sound and reproducible assessment strategies are essential for validating and optimizing nanotechnology-based approaches. Such methodologies will not only ensure that scientific advancements translate into clinically relevant and reproducible outcomes but also help minimize bottlenecks in the clinical translation process.

A major opportunity for advancement lies in the inherently multidisciplinary nature of this field. Cross-disciplinary collaboration holds the potential to synthesize diverse findings and strengthen future directions. While research gaps are closing in the body of knowledge, new therapeutic opportunities are emerging, creating pathways for innovation in CNS therapeutics.

## Conclusions

Research on the BBB has advanced significantly, laying the foundation for new therapeutic strategies. In this context, nanotechnology emerges as an innovative tool to enhance drug delivery to the CNS. However, despite notable progress, key challenges remain. A deeper understanding of BBB physiology and its disease-related changes, the refinement of transport-targeting mechanisms, the development of physiologically relevant evaluation models, and the establishment of clear regulatory pathways are all essential for achieving clinically viable solutions.

This review provides a comprehensive overview of the foundational elements that can shape strategies for overcoming the BBB. By integrating insights from lipid-based nanotechnology, functionalization techniques, and permeability models, it identifies critical research gaps and opportunities. Addressing these challenges through interdisciplinary collaboration will be essential for translating promising nanomedicine-based CNS therapies from the bench to the bedside. Moving forward, the integration of fundamental research, applied nanomedicine, and regulatory science will be crucial—not only to unlocking the full potential of CNS-targeted therapies but also for opening new pathways across the BBB for the treatment of neurological diseases, psychiatric disorders, and brain tumours.

## Abbreviations

BBB: Blood-brain barrier
BL: Basal lamina
ECs: Endothelial cells
LNPs: Lipid nanoparticles
NVU: Neurovascular unit
P-gp: P-glycoprotein
RVG: Rabies virus glycoprotein
γ-GT: γ-Glutamyl Transpeptidase
BCRP: Breast Cancer Resistance Protein
CNS: Central Nervous System
iPSCs: Induced Pluripotent Stem Cells
LRP1: Low-density lipoprotein receptor-related protein 1
OoC: Organ-on-a-chip
TEER: TransEndothelial Electric Resistance
TJ: Tight junctions
